# Occurrence and genetic characterization of *Echinococcus granulosus* sensu *lato* from domestic animals in Central Iran

**DOI:** 10.1186/s12917-021-03131-1

**Published:** 2022-01-07

**Authors:** Bahador Hajimohammadi, Abdolhossein Dalimi, Gilda Eslami, Salman Ahmadian, Sajad Zandi, Ahmad Baghbani, Saeedeh Sadat Hosseini, Vahideh Askari, Maryam Sheykhzadegan, Mehrnoosh Nabizadeh Ardekani, Mohammad Javad Boozhmehrani, Mohammad Javad Ranjbar, Hamed Ghoshouni, Mahmood Vakili

**Affiliations:** 1 Research Center for Food Hygiene and Safety, Shahid Sadoughi University of Medical Sciences, Yazd, Iran; 2 Department of Food Hygiene and Safety, School of Public Health, Shahid Sadoughi University of Medical Sciences, Yazd, Iran; 3 Department of Parasitology, Faculty of Medical Sciences, Tarbiat Modares University, Tehran, Iran; 4 Department of Parasitology and Mycology, School of Medicine, Shahid Sadoughi University of Medical Sciences, Yazd, Iran; 5 Inspection of the Chief Health Officer (Meat Inspector), Yazd, Iran; 6 Department of Community and Preventive Medicine, Health Monitoring Research Center, School of Medicine, Shahid Sadoughi University of Medical Sciences, Yazd, Iran

**Keywords:** *Echinococcus granulosus* sensu *lato*, Livestock, *Cytochrome C oxidase subunit* 1, Food safety, Genotypes

## Abstract

**Background:**

The species complex of *Echinococcus granulosus* sensu *lato* (*s.l.*) causes cystic echinococcosis distributed worldwide. There is no genotype information from hydatid cysts in the intermediate hosts in Central Iran. Therefore, in this study, we analyzed the hydatid cysts in livestock slaughtered in an abattoir in this region. Six hundred fifty-seven hydatid cysts were isolated from 97 animals, including sheep, cattle, camels, and goats slaughtered in Yazd abattoir from September 2018 to January 2020. The demographic data was collected as well as cyst location, fertility, and viability. Out of 657 samples, 164 samples were genotyped. Then, phylogenetic analysis was performed using MEGAX. Statistical analyses were done using SPSS version 16.0 by chi-square with a significant difference of less than 0.05.

**Results:**

Out of 164 samples, the G1-G3 complex genotype had the most frequency in samples, with 135 cases recognized. The G6/G7 was observed in 19 isolates and G5 was reported in nine samples. One sample was detected as *Taenia hydatigena.*

**Conclusions:**

This study showed that G1-G3 and G6/G7 genotypes were presented in all animals, but G5 was reported only in cattle, goats, and camels. It is the first molecular identification of cystic echinococcosis in Central Iran. Hence, reporting G5 in livestock in this area should be considered due to transmission to humans.

## Background

The species complex of *Echinococcus granulosus* sensu *lato* (*s.l.*) is a parasite causing cystic echinococcosis (CE) [1]. Echinococcosis is one of the most important tropical diseases worldwide distribution due to its remarkable economic damages [2]. Livestock such as sheep, cattle, goats, and camels acts as intermediate hosts, which harbor metacestodes in the liver, lung, and other internal organs. Besides, adult tapeworms are in the intestine of wild and domestic canids that serve as definitive hosts [3].

*E. granulosus s.l.* is composed of distinct genotypes that are divided into *E. granulosus* sensu stricto (genotype G1–G3 exist in sheep and buffalos), *E. equinus* (G4 found in horses), *E. ortleppi* (G5 in cattle), and *E. canadensis* (G6/G7, G8, and G10) [4]. The most eminent genotype in the world is G1 (sheep) [5].

The echinococcosis has been reported in Australia, northern and eastern Africa, Central Asia, and some Mediterranean countries [6, 7]. Additionally, infection in livestock and ruminants has been described in different parts of Iran [8]. Several slaughterhouses in Iran have a total prevalence of 5–72% in sheep, 11.4–70% in camels, 3.5–38% in cattle, and 1.7–20% in goats [9–11].

Based on the available evidence, some genotypes have higher pathogenicity for humans than others, so the determination of *E. granulosus s.l.* genotype is essential for disease control, drug reactions, and prevention in different geographical areas [12–14]. Based on sequence analysis and further techniques conducted in Iran and other countries, the dominant genotypes include G1-G3 and G6/G7 [15]. No study has been conducted regarding the molecular identification of *E. granulosus s.l.* in Central Iran. To increase knowledge of the genotype spectrum involving CE in Central Iran, the present study was conducted to evaluate the genetic variation of *Echinococcus granulosus s.l.* in livestock slaughtered in this area.

## Results

The hydatid cysts included in this study were obtained from livestock, including sheep, camels, goats, and cattle. The cyst locations were in both animal liver and lung with the rate of 38.1% in sheep with both lung and liver infection; 38.9% of camels had both lung and liver infection, 25% of cattle had both lung and liver infection, and 50% of goats were infected in both lung and liver. One sample was detected as *Taenia hydatigena.* The identified *E. granulosus s.l.* genotypes were G1-G3, G6/G7, and G5. One crucial piece of data shown in this study was that more than one genotype was identified in one animal species infected with hydatid cysts. One goat had two cysts in the lung with two different genotypes of G1-G3 and G6/G7. In cattle, two had cysts in their lungs with two different genotypes of G1-G3 and G5 and one had co-infection of two genotypes of G1-G3 and G6/G7 in the lung. In camels, four animals had co-infection of G1-G3 and G6/G6 genotypes, two had co-infection of G1-G3 and G5 genotypes, and one had three genotypes of G1-G3, G6/G7, and G5. In sheep, co-infection has not been recorded.

### Prevalence of infection per sex

**Fig. 1:**
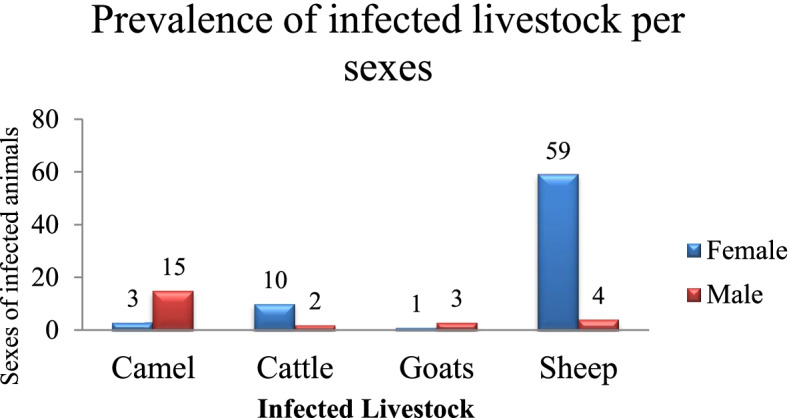
The prevalence *Echinoccus granulosus s.l.* per sexes in slaughtered livestock

Among 97 infected animals, 75.3% (73/97) were female, including 1.4% (1/73) of goats, 80.8% (59/73) of sheep, 4.1% (3/73) of camels, and 13.7% (10/73) of cattle. The male with echinococcosis was 24.7% (24/97) comprising 16.7% (4/24) sheep, 12.5% (3/24) goats, 8.3% (2/24) cattle, and 62.5% (15/24) camels (Fig. 1). Regarding infection in each animal species per sex, it was 93.7% (59/63) female and 6.3% (4/63) male in sheep, 25% (1/4) female and 75% (3/4) male in goats, 83.3% (10/12) female and 16.7% (2/12) male in cattle, and 16.7% (3/18) female and 83.3% (15/18) male in camels.

### Prevalence of infection per age

**Fig. 2:**
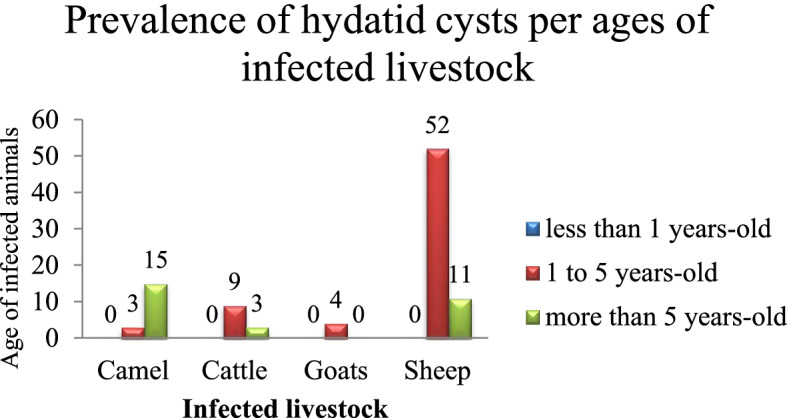
The prevalence *Echinoccus granulosus s.l.* per ages in slaughtered livestock

Out of 97 infected animals, 70.1% (68/97) were 1 to 5 years old, including 76.5% (52/68) of sheep, 5.9% (4/68) of goats, 13.2% (9/68) of cattle, and 4.4% (3/68) of camels; and 29.9% (29/97) were more than 5 years old comprising 37.9% (11/29) of sheep, 10.3% (3/29) of cattle, and 51.8% (15/29) of camels. The most infected sheep, goats, and cattle were 1 to 5 years old, and the most infected camels were more than 5 years old (Fig. 2). Regarding each animal species, 82.5% (52/63) of sheep were 1 to 5 years old, and 17.5% (11/63) were more than 5 years old; all goats were 1 to 5 years old; 75% (9/12) of cattle were 1 to 5 years old, and 25% (3/12) were more than 5 years old; and finally, 16.7% (3/18) of camels were 1 to 5 years old, and 83.3% (15/18) were more than 5 years old. None of the infected animals was less than 1 year old.

### Prevalence of infection per cyst location

**Fig. 3:**
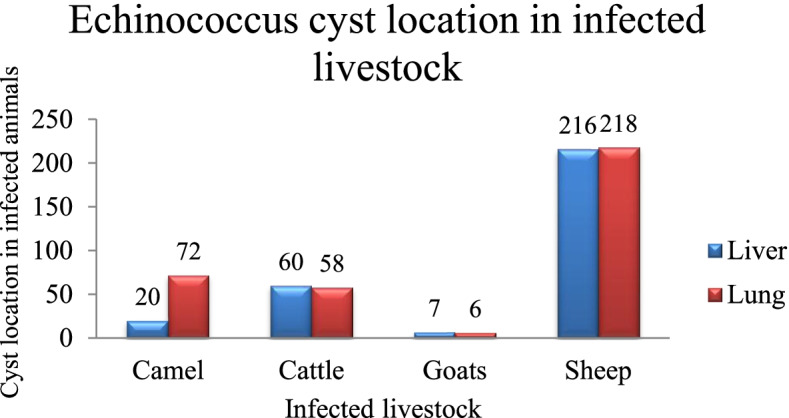
The prevalence *Echinoccus granulosus s.l.* per cyst locations in slaughtered livestock

From 657 hydatid cysts obtained from 97 animals, the distribution of cysts in the lung and liver was almost the same in sheep, goats, and cattle. However, in camels, the lung had more cysts than the liver. Out of 657 cysts, 46.1% (303/657) samples were obtained from the animals’ liver, including 71.3% (216/303) of sheep, 19.8% (60/303) of cattle, 6.6% (20/303) of camels, and 2.3% (7/303) of goats. The hydatid cysts in animals’ lung were 53.9% (354/657), including 61.6% (218/354) in sheep, 20.3% (72/354) in camel, 16.4% (58/354) in cattle, and 1.7% (6/354) in goats (Fig. 3). Regarding the animal species and the cysts’ location, its prevalence in the goat liver was 53.8%, and in the goat lung was 46.2%. Besides, it was 49.8% in sheep liver and 50.2% in sheep lung. This presence was 50.8% in cattle liver and 49.2% in cattle lung; and 21.7% in camel liver and 78.3% in camel lung.

### Fertility and viability

**Fig. 4:**
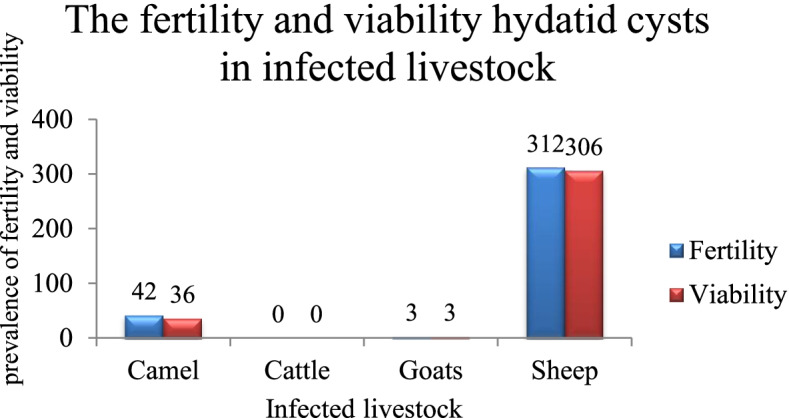
The fertility and viability of *Echinoccus granulosus s.l.* cysts per slaughtered livestock

Out of 657 samples, 357 (54.3%) were fertile, including 87.4% (312/357) of sheep, 0.8% (3/357) of goats, and 11.8% (42/357) of camels. None of the cysts in camels were fertile. Regarding each animal species, the rate of hydatid cysts fertility in sheep was 71.9% (312/434), it was 23% (3/13) in goats and 45.7% (42/92) in camels. Out of 357 fertile cysts in animals, 96.6% (345/357) had viability, including 88.7% (306/345) in sheep, 0.9% (3/345) in goats, and 10.4% (36/345) in camels. Respective in each animal, the viability rate of the fertile cysts was 98.1% (306/312) in sheep and 85.7% (36/42) in camels. All fertile cysts in goats had viability (Fig. 4).

The descriptive analysis showed that 75.3% of the infected animals were female. The chi-square analysis indicated that infection rate and sex had a significant relationship (*p =* 0.00001). The infection rate at the age of 1 to 5 was 70.1%, and the remaining was regarding the animals more than 5 years old. The statistical analysis showed significant differences among the age groups concerning the infection rate (*p* = 0.00001). The location of hydatid cysts in animals differed, with 46.1% in the liver and 53.9% in the lung. The analysis revealed statistical significance between infection rate and cyst location in animals (*p* = 0.000011). We showed that 54.3% of hydatid cysts in animals were fertile, of which 87.4% were regarding cysts in sheep, following 11.7% in camels and 0.9% in goats. Among the fertile cysts, 96.6% were viable after staining with eosin 0.1%, of which 88.7% were related to sheep, 10.4% to camels, and 0.9% to goats. Statistical analysis showed a significant relationship between viability and fertility with animal kinds (*p* = 0.00001).

### Molecular detection

**Fig. 5:**
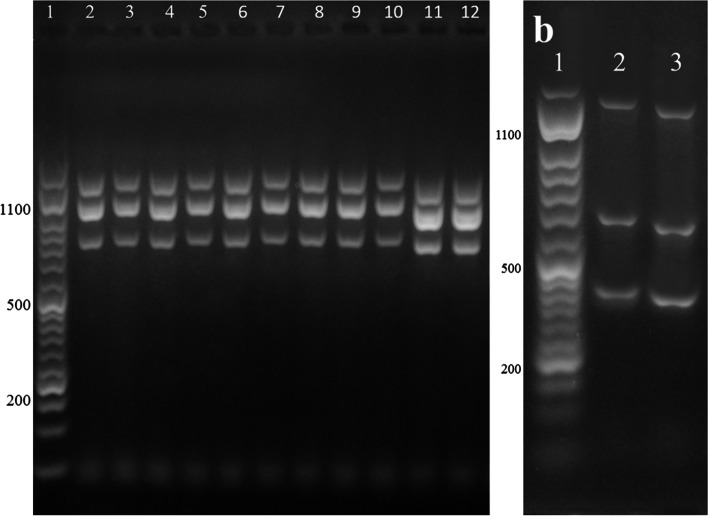
Agarose gel electrophoresis of multiplex PCR analysis. **a** Lane 1: 50 bp DNA ladder, lanes 2–12: G1-G3 genotypes. **b** Lane 1: 50 bp DNA ladder, lanes 2–3: G6/G7 genotypes. The fragments of 1232 bp is specific for the *Echinococcus* genus. The bands with the size of 1001 and 706 are related to G1-G3 gentotypes; the fragments of 617 and 339 bp are corresponding to G6/G7 genotypes

**Fig. 6:**
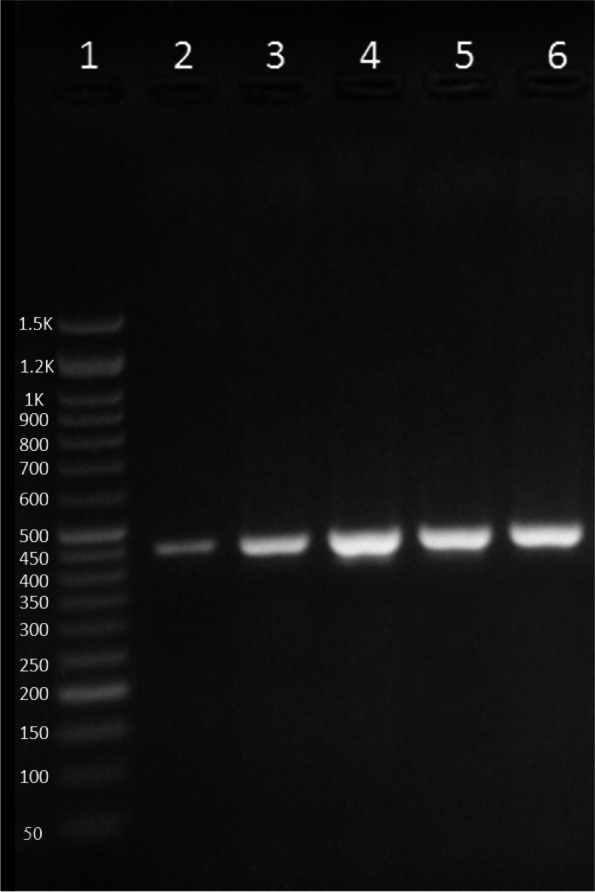
Agarose gel electrophoresis for *cox* 1 amplification analysis. Lane 1: 50 bp DNA ladder, lanes 2–6: amplified *cox* 1 region in the samples. The expected amplicon size is 450 bp in length

Out of 657 hydatid cysts, 164 isolates were randomly selected and assessed for molecular genotyping using multiplex PCR (Fig. 5). Finally, out of 164 isolates, 59 were randomly selected for sequencing, and they were amplified by PCR using the specific primer pair of the gene target of *cytochrome c oxidase subunit* 1 (*cox* 1) resulted in the size of the amplicon of 450 bp in length (Fig. 6). The sequences were submitted to NCBI, GenBank (Table 1).

**Table 1:** The sequences used in the study

Accession Number	Description
MW553931 (G1)	*Echinococcus granulosus* isolate S1K1 cytochrome c oxidase subunit I (*COX1*) gene
MW724484 (G1)	*Echinococcus granulosus* isolate S1K2 cytochrome c oxidase subunit I (*COX1*) gene
MW724481 (G1)	*Echinococcus granulosus* isolate S2K11 cytochrome c oxidase subunit I (*COX1*) gene
MW509612 (G1)	*Echinococcus granulosus* isolate S1k3 cytochrome c oxidase subunit I (*COX1*) gene
MW674790 (G1)	*Echinococcus granulosus* isolate S1K4 cytochrome c oxidase subunit I (*COX1*) gene
MW509613 (G1)	*Echinococcus granulosus* isolate S2K1 cytochrome c oxidase subunit I (*COX1*) gene
MW665457 (G1)	*Echinococcus granulosus* isolate S2K3 cytochrome c oxidase subunit I (*COX1*) gene
MW666055	*Taenia hydatigena* isolate S3K1 cytochrome c oxidase subunit I (*COX1*) gene
MW571043 (G1)	*Echinococcus granulosus* isolate S4R1 cytochrome c oxidase subunit I (*COX1*) gene
MW672137 (G1)	*Echinococcus granulosus* isolate S6R2 cytochrome c oxidase subunit I (*COX1*) gene
MW666181 (G1)	*Echinococcus granulosus* isolate S6R3 cytochrome c oxidase subunit I (*COX1*) gene
MW672197 (G1)	*Echinococcus granulosus* isolate S6R10 cytochrome c oxidase subunit I (*COX1*) gene
MW666108 (G1)	*Echinococcus granulosus* isolate S7K3 cytochrome c oxidase subunit I (*COX1*) gene
MW676786 (G1)	*Echinococcus granulosus* isolate S7K5 cytochrome c oxidase subunit I (*COX1*) gene
MW567930 (G1)	*Echinococcus granulosus* isolate S7K6 cytochrome c oxidase subunit I (*COX1*) gene
MW898297 (G1)	*Echinococcus granulosus* isolate S9R2 cytochrome c oxidase subunit I (*COX1*) gene
MW724480 (G1)	*Echinococcus granulosus* isolate S10R1 cytochrome c oxidase subunit I (*COX1*) gene
MW672209 (G3)	*Echinococcus granulosus* isolate S16R1 cytochrome c oxidase subunit I (*COX1*) gene
MW563951 (G1)	*Echinococcus granulosus* isolate S18R1 cytochrome c oxidase subunit I (*COX1*) gene
MW563953 (G1)	*Echinococcus granulosus* isolate S19R3 cytochrome c oxidase subunit I (*COX1*) gene
MW563946 (G3)	*Echinococcus granulosus* isolate S23R1 cytochrome c oxidase subunit I (*COX1*) gene
MW564021 (G1)	*Echinococcus granulosus* isolate S24R2 cytochrome c oxidase subunit I (*COX1*) gene
MW564030 (G1)	*Echinococcus granulosus* isolate S26R1 cytochrome c oxidase subunit I (*COX1*) gene
MW564032 (G1)	*Echinococcus granulosus* isolate S31K1 cytochrome c oxidase subunit I (*COX1*) gene
MW898298 (G1)	*Echinococcus granulosus* isolate S34K1 cytochrome c oxidase subunit I (*COX1*) gene
MW564076 (G1)	*Echinococcus granulosus* isolate S38K1 cytochrome c oxidase subunit I (*COX1*) gene
MW564079 (G1)	*Echinococcus granulosus* isolate S39K1 cytochrome c oxidase subunit I (*COX1*) gene
MW566585 (G1)	*Echinococcus granulosus* isolate S40K1 cytochrome c oxidase subunit I (*COX1*) gene
MW564207 (G1)	*Echinococcus granulosus* isolate S41R1 cytochrome c oxidase subunit I (*COX1*) gene
MW566168 (G1)	*Echinococcus granulosus* isolate S42K2 cytochrome c oxidase subunit I (*COX1*) gene
MW666128 (G1)	*Echinococcus granulosus* isolate S43K1 cytochrome c oxidase subunit I (*COX1*) gene
MW567458 (G1)	*Echinococcus granulosus* isolate S48R1 cytochrome c oxidase subunit I (*COX1*) gene
MW724526 (G1)	*Echinococcus granulosus* isolate S51K2 cytochrome c oxidase subunit I (*COX1*) gene
MW672317 (G1)	*Echinococcus granulosus* isolate S52K1 cytochrome c oxidase subunit I (*COX1*) gene
MW566173 (G1)	*Echinococcus granulosus* isolate S56K1 cytochrome c oxidase subunit I (*COX1*) gene
MW666180 (G1)	*Echinococcus granulosus* isolate S57K2 cytochrome c oxidase subunit I (*COX1*) gene
MW683516 (G1)	*Echinococcus granulosus* isolate B4K1 cytochrome c oxidase subunit I (*COX1*) gene
MW683965 (G1)	*Echinococcus granulosus* isolate B5K2 cytochrome c oxidase subunit I (*COX1*) gene
MW509614 (G1)	*Echinococcus granulosus* isolate B5k6 cytochrome c oxidase subunit I (*COX1*) gene
MW567466 (G1)	*Echinococcus granulosus* isolate B7R1 cytochrome c oxidase subunit I (*COX1*) gene
MW567290 (G5)	*Echinococcus granulosus* isolate B7R3 cytochrome c oxidase subunit I (*COX1*) gene
MW567132 (G1)	*Echinococcus granulosus* isolate B7R5 cytochrome c oxidase subunit I (*COX1*) gene
MW567286 (G1)	*Echinococcus granulosus* isolate B8R3 cytochrome c oxidase subunit I (*COX1*) gene
MW665388 (G1)	*Echinococcus granulosus* isolate B8R4 cytochrome c oxidase subunit I (*COX1*) gene
MW546059 (G5)	*Echinococcus granulosus* isolate C2R3 cytochrome c oxidase subunit I (*COX1*) gene
MW665386 (G1)	*Echinococcus granulosus* isolate C3K2 cytochrome c oxidase subunit I (*COX1*) gene
MW546060 (G1)	*Echinococcus granulosus* isolate C3R2 cytochrome c oxidase subunit I (*COX1*) gene
MW665389 (G1)	*Echinococcus granulosus* isolate C3K5 cytochrome c oxidase subunit I (*COX1*) gene
MW549013 (G6)	*Echinococcus granulosus* isolate C3R5 cytochrome c oxidase subunit I (*COX1*) gene
MW549010 (G5)	*Echinococcus granulosus* isolate C3R7 cytochrome c oxidase subunit I (*COX1*) gene
MW665390 (G5)	*Echinococcus granulosus* isolate C3R9 cytochrome c oxidase subunit I (*COX1*) gene
MW671557 (G1)	*Echinococcus granulosus* isolate C4K1 cytochrome c oxidase subunit I (*COX1*) gene
MW665387 (G5)	*Echinococcus granulosus* isolate C4R7 cytochrome c oxidase subunit I (*COX1*) gene
MW567459 (G6)	*Echinococcus granulosus* isolate G2R1 cytochrome c oxidase subunit I (*COX1*) gene
MW549002 (G1)	*Echinococcus granulosus* isolate G3R1 cytochrome c oxidase subunit I (*COX1*) gene
MW549003 (G6/G7)	*Echinococcus granulosus* isolate G3R2 cytochrome c oxidase subunit I (*COX1*) gene
MW549009 (G1)	*Echinococcus granulosus* isolate G3K3 cytochrome c oxidase subunit I (*COX1*) gene
MW676785 (G1)	*Echinococcus granulosus* isolate G4R1 cytochrome c oxidase subunit I (*COX1*) gene
MW564020 (G1)	*Echinococcus granulosus* isolate G4R3 cytochrome c oxidase subunit I (*COX1*) gene
**Reference**	
NC_038228.1	*Echinococcus granulosus* sensu *lato* genotype G7 isolate 27 mitochondrion, complete genome
NC_038227.1	*Echinococcus granulosus* sensu *lato* genotype G6 isolate 1 mitochondrion, complete genome
NC 011122.1	*Echinococcus ortleppi* mitochondrion, complete genome
NC_044548.1	*Echinococcus granulosus* mitochondrion, complete genome
NC_021144.1	*Echinococcus felidis* mitochondrial DNA, complete genome, sample code: EfelUganda
NC_009462.1	*Echinococcus vogeli* mitochondrion, complete genome
NC_009460.1	*Echinococcus shiquicus* mitochondrion, complete genome
NC_009461.1	*Echinococcus oligarthrus* mitochondrion, complete genome
NC_000928.2	*Echinococcus multilocularis* mitochondrion, complete genome
NC_020374.1	*Echinococcus equinus* mitochondrion, complete genome
GQ228819.1	*Taenia hydatigena* mitochondrion, complete genome

The genotyping of the 164 samples showed that one isolate was *Taenia hydatigena*. The remaining 163 samples were 82.8% (135/163) G1-G3 genotypes, including 58.5% (79/135) in sheep, 24.5% (33/135) in camels, 12.6% (17/135) in cattle, and 4.4% (6/135) in goats; 11.7% (19/163) samples were G6/G7, including 52.7% (10/19) in camels, 21% (4/19) in sheep, 15.8% (3/19) in cattle, and 10.5% (2/19) in goats; and finally, 5.5% (9/163) had G5 genotype, including 44.4% (4/9) in camels, 44.4% (4/9) in cattle, and 11.2% (1/9) in goats. Sequence analysis showed that among *E. granulosus s.s.* identified in this study, two isolates were identified only as G3 that were in sheep lung. No G5 genotype was found in sheep.

Out of 135 hydatid cysts with G1-G3 genotypes, 54.1% (73/135) samples were in the lung and 45.9% (62/135) were in the liver. The G6/G7 genotype cysts were in the lung and liver, with the rate of 78.9% (15/19) and 21.1% (4/19), respectively. The cysts with the genotype of G5 were in the lung and liver with the rate of 88.9% (8/9) and 11.1% (1/9), respectively. The distribution of the different genotypes was variously based on the location of each animal (Table 2); however, the cyst location in each animal had no significant relationship with genotypes.

**Table 2:** The distribution of the different genotypes based on the cyst location of each cyst

	Genotypes			
	G1-G3	G6/G7	G5	*P-value*
Camel lung	23	8	4	> 0.05
Camel liver	10	2	0	
Sheep liver	41	1	0	> 0.05
Sheep lung	38	3	0	
Goat lung	4	2	0	> 0.05
Goat liver	2	0	1	
Cattle lung	8	2	4	> 0.05
Cattle liver	9	1	0	

Regarding fertility and genotypes, 50.1% (73/135) of animal cysts with G1-G3 genotypes were fertile, of which 95.9% (70/73) had viability. The fertility rate for G6/G7 genotype was 31.6% (6/19), of which 83.3% (5/6) had viability. In G5 hydatid cysts, 15.8% (3/19) were fertile, of which 66.7% (2/3) had viability. Regarding chi-square statistics with *p* = 0.002562, the fertility rate is significantly concerning genotypes. The viability rate was related to genotypes (*p =* 0.034222).

### Phylogeny analysis

**Fig. 7:**
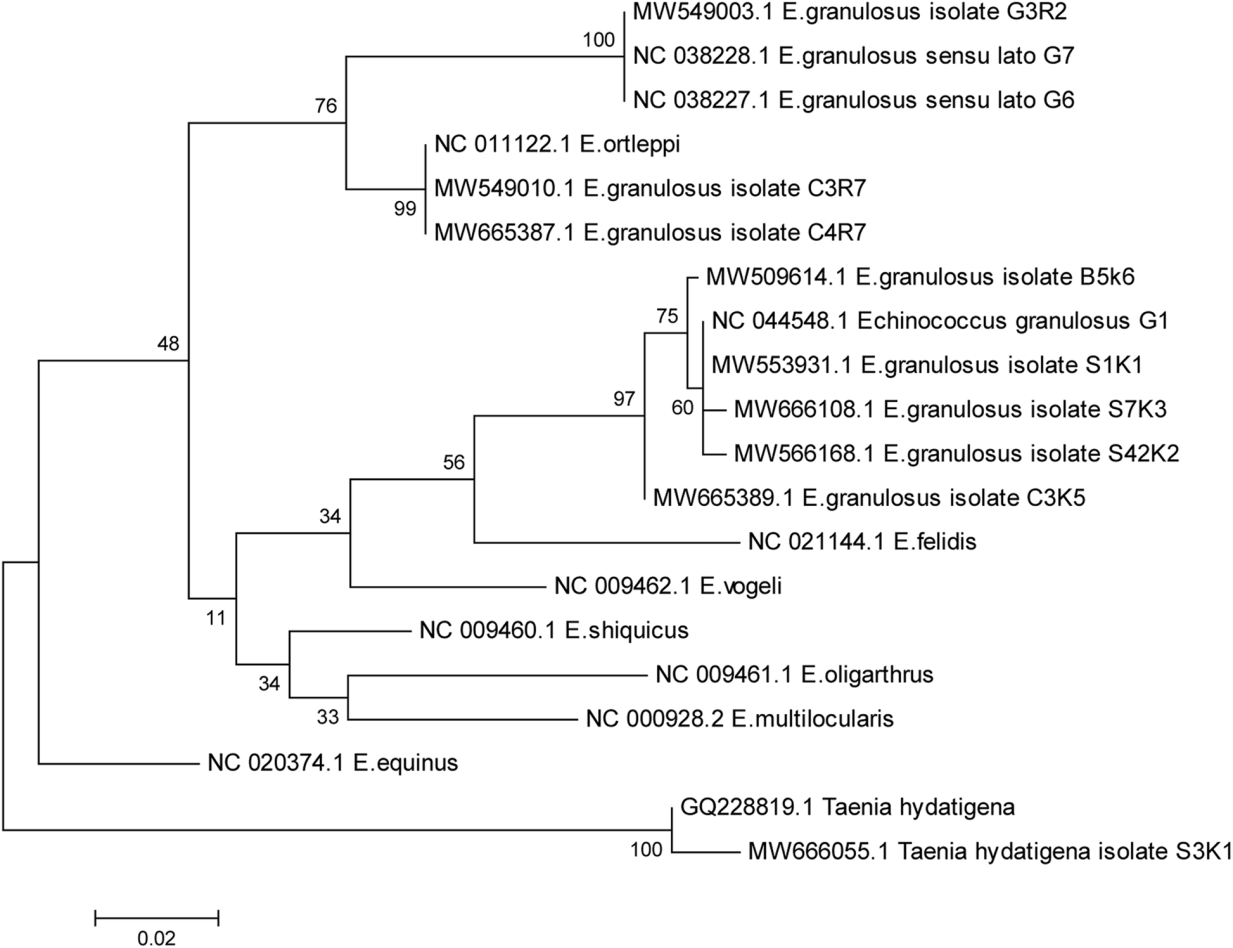
Molecular phylogenetic analysis of *Echinococcus granulosus* isolates based on *cox1* sequences. The evolutionary history was inferred by using the Maximum Likelihood method based on the Tamura-Nei model. The analysis involved 20 nucleotide sequences. All positions containing gaps and missing data were eliminated. Evolutionary analyses were conducted in MEGAX

In the Maximum Likelihood (ML) consensus tree obtained, all the specimens here analyzed clustered together with G1-G3 reference strains, demonstrating the circulation of the *E. granulosus s.s.* in the area (Fig. 7).

## Discussion

In this study, 657 hydatid cyst samples were isolated from 63 sheep, 18 camels, 12 cattle, and 4 goats. In 97 infected animals, 73 (75.3%) were females, even though the prevalence of infection was different in animal species (*p* = 0.00001). A higher prevalence in females was also described in several studies [16–23]. Female animals are at higher risk of CE infection because females are kept longer for milk production and reproductive functions [18]. In addition, females are raised near houses for milking, making them more exposed to infected dogs [19]. The relationship between age and the infected animals was significant in this study (*p* = 0.00001). The infection rate was high in animals aged between 1 and 5 years, except for camels where high infection rates were seen in animals older than 5 years. The increasing prevalence rate with age is in agreement with other studies carried out in several countries [9, 17–19, 24–30]. The causes of infection in older animals are longer duration of exposure and more time for cysts to grow in size for better diagnosis [18]. The cyst develops in camels after the age of 3 [31]. Therefore, we showed that the infection rate is related to the sex and age of animals. All cysts from infected animals were isolated from the lung and liver. We presented that the cyst location had a significant relationship with the infected animal species. Some other results from different studies also show a vast distribution of the cyst in animal organs. Azami et al. [16] reported that the highest and lowest cysts were in sheep lungs (27.1%) and goat liver (11.6%), respectively. In Pakistan, Ehsan et al. [18] demonstrated cysts in cattle, with 8.65% in the liver and 4.80% in the lung. Haleem et al. [19] reported that in Pakistan, the prevalence of hydatid cysts in the liver and lung was 63.49 and 23.80%, respectively, including 69 cattle livers, 9 cattle lungs, 12 sheep livers, 14 sheep lungs, 3 goat livers, and 1 goat lung [19]. In another study in Pakistan, hydatid cysts were distributed in the liver and lung of various animals, including sheep with 67.81% in liver and 32.19% in lung, goats with 66.18% in liver and 32.60 in the lung, cattle with 84.51% in liver and 15.48% in lung, and camels with 83.33% in liver and 16.66% in the lung [32]. In the other study in Pakistan, the infection rates were 46.74% in sheep liver, 23.28% in goat liver, 17.37% in sheep lung, and 13.68% in goat lung [33]. Singh et al. [34] reported liver and lung infection rates with 49.66 and 36.179%, respectively, in India’s cattle, buffaloes, sheep, and goats. Elmajdoub and Rahman [35] in Libya demonstrated that among 372 samples in different organs, including 135 sheep livers, 105 sheep lungs, 28 camel livers, 64 camel lungs, 6 cattle livers, and 6 lung cattle. In a study carried out in north Ethiopia, hydatid cysts in cattle were 13% in the lung and 8.1% in the liver [36]. The organs’ involvement is because passing the oncosphere is carried out via portal vein flow into the liver and other organs [37].

A high fertility rate was observed in cysts isolated from sheep, followed by goats and camels. No fertility was reported in the samples from cattle. The fertility rate of hydatid cysts was higher in the liver of sheep, whereas in camels and goat, fertile cysts were mostly found in lungs. Some studies have reported more fertility in the liver [16, 20, 38–40], and some studies reported more fertility in the lung [41–44]. In this study, it was found that none of the cysts from the cattle were fertile. This result is in agreement with some other studies [30, 45, 46]. No fertility in the hydatid cysts of the cattle reveals that this intermediate host would not be considered suitable, and therefore, cattle are a dead-end host [47]. Hence, understanding the fertility and viability of the hydatid cysts in the intermediate host is critical because livestock play as the main source of infection transmission of the final host by consuming fertile cysts. The fertility of the hydatid cyst is different in various geographical situations, animal species, locations, size, and genotype of the cyst [48]. There are many reports about the infertility rate of the hydatid cysts in various genotypes and animal species in different regions [36, 49], indicating that fertility depends on the genotype. The fertility rate may be different in various hosts due to the immunological response in the hosts [50]. In our study, high rate of cyst viability was observed in sheep, goats, and camel, in agreement with other studies [16, 19, 51].

This study was the first molecular detection and identification of *E. granulosus s.l.* in slaughtered livestock in a desert area in Central Iran. However, more molecular epidemiological studies have been done on hydatid cysts in intermediate hosts in some other areas of Iran [8]. The only limitation in this study is that the molecular characterization was not performed on all hydatid cysts. Genotyping analysis in this study showed that *E. granulosus s.l.* G1-G3 genotypes were the most frequent in this area in all animals.

Out of 135 isolates belonging to *E. granulosus s.s.* (G1-G3), 58.5% (79/135) were found in sheep, 24.5% (33/135) in camels, 12.6% (17/135) in cattle, and 4.4% (6/135) in goats. Among *E. granulosus s.s.* identified in this study, two isolates were identified only as G3. This result agrees with other studies conducted in different regions of Iran [52–59]. Sharbatkhori et al. [60] reported 78.3% G1 genotype among hydatid cysts obtained from livestock in Golestan Province. Pezeshki et al. [61] also claimed 92% G1 in the hydatid cysts isolated from domestic animals in Ardabil Province, northwestern Iran. Whereas, Nematdoost et al. [62] reported 7.2% G3 genotype in hydatid cysts collected from livestock. Some studies have also reported G3 genotype in Iran and other countries [56, 57, 59, 60, 63–67]. Also, among *E. granulosus s.l.*, *E. canadensis* (G6/G7) and *E. ortleppi* (G5) were reported in slaughtered livestock with a prevalence of 11.7 and 5.5%, respectively. From 19 isolates belonging to *E. canadensis* (G6/G7), 52.7% (10/19) were found in camels, 21% (4/19) in sheep, 10.5% (2/19) in goats, and 15.8% (3/19) in cattle. Thus, it seems that cattle, camels, sheep, and goats are involved in the life cycle in Iran. In addition, there are some reports from different livestock worldwide. Rajabloo et al. [68] reported the G6 genotype in goats in Iran. Kesik et al. [69] reported one sample with the G6 genotype obtained from a camel in Turkey. There are also some reports of G6 genotype in goat in Argentina [70, 71]. The main intermediate host for the G6 genotype is the camel. However, it seems that camels living with other livestock may expose to other genotypes through interaction with dogs as definitive hosts living near them. There are some reports of G6 in humans worldwide. The first report of *E. Canadensis* (G6/G7) in humans found in Pakistan shows the importance of this genotype in clinical aspects [72], even though it was thought that the G6 genotype might be less infective to humans [73]. However, it is considered as the second most important causative agent of CE after *E. granulosus s.s* [74]. In South America, Africa, and Asia; *E. granulosus s.l.* G6 genotype infects humans [73]. Simsek and Kaplan [75] also reported two cases of human infection with the G6 genotype in Turkey.

*E. ortleppi* (G5) was not reported from sheep, but it was identified from nine cysts, one belonging to goats (11.2%), four to camels (44.4%), and four to cattle (44.4%). This result is consistent with other studies done in different regions of Iran [52–58]. As mentioned above, *E. ortleppi* (G5) was reported in 5.5% (9/163) of *E. granulosus s.l.* cysts from goats, camels, and cattle that is less than the study by Nematdoost et al. [62] reported G5 in 5/45 (11.1%) of cattle. Some other studies reported G5 genotype in buffaloes [76], goats [77], and camels [77–79]. Moreover, some studies reported G5 genotype in sheep [77, 80] that is opposite to our results. In this study, 75% (3/4) of hydatid cysts found in camels with G5 genotype were fertile, whereas G5 in goats and cattle were infertile. The result concerning the G5 genotype showing no fertility in cattle agrees with the study conducted in Pakistan [81]. However, Monteiro et al. [82] reported the frequency of 43.4% G5 genotype for fertile cysts in the lung of cattle in Brazil.

## Conclusions

To the best of our knowledge, the molecular identification of hydatid cysts was performed for the first time in the livestock slaughtered in Central Iran. The genotypes of G1-G3 and G6/G7 exist in all livestock being studied, but G5 was reported only in cattle, goats, and camels. Therefore, the dominant genotypes in Central Iran are G1-G3 and then G6/G7, which should be considered a high-priority public health concern.

## Methods

In this study, 97 slaughtered animals with echinococcosis from Yazd abattoir were included to assess the variables of sex, age, cyst location, fertility, viability of protoscoleces, and genotyping. In this study, we used the discarded organs with hydatid cysts.

### Ethical approval

All experiments were ethically performed following standard protocols approved by the Ethics Committee of Shahid Sadoughi University of Medical Sciences, Yazd, Iran (Approval ID: IR. SSU. SPH.REC. 1398.068).

### Sample collection

In the current study, 657 hydatid cysts were isolated from 63 sheep, 12 cattle, 18 camels, and 4 goats, comprising 434 samples from sheep, 118 samples from cattle, 13 samples from goats, and 92 from camels from September 2018 to January 2020 from slaughtered animals during post-mortem inspection from an abattoir in Yazd Province, Central Iran. Collected cysts from lungs and livers were transported to the Research Center of Food Hygiene and Safety, Shahid Sadoughi University of Medical Sciences, Yazd, Iran. The sex and age of each animal were recorded by a veterinarian in the slaughterhouse. The eruption of permanent incisor teeth was the main criteria used to determine the age of the animals [83].

### Viability and fertility

After sterilizing the cyst surface with 70% alcohol, the cyst fluid was aspirated and centrifuged at 500 xg for 60 s. For fertility assessment, the pellet was analyzed for protoscoleces present using a light microscope. Cysts without any protoscoleces were detected as sterile. The viability of the protoscoleces was evaluated using 0.1% eosin staining. The nonviable protoscoleces were stained in red.

### DNA extraction

Protoscoleces (if presented) and germ layers were used for genotype analysis. The protoscoleces were washed with phosphate-buffered solution (PBS) in triplicate. According to the manufacturer’s instructions, the genomic DNA (gDNA) was extracted using a Tissue DNA Extraction Kit (GeneAll, South Korea). The quantity of the extracted DNA samples was analyzed using a NanoDrop device (Thermo Fisher Scientific, Massachusetts, USA) and stored at − 20 °C until the next analysis.

### Molecular detection

Characteristics of primers used for multiplex PCR are shown in Table 3. PCR reaction was conducted in a 50 μl final volume containing 50–100 ng of gDNA, 0.2 mM dNTPs, 2 mM MgCl_2_, and different concentrations of each primer are described in Tables 3, 1.5 U of *Taq* DNA polymerase, and sterile ddH_2_O up the final volume. The cycling conditions were as follows: an initial denaturation step at 94 °C for 3 min; 30 cycles of 94 °C for 30 s, 56 °C for 30 s, 72 °C for 1 min; and a final extension for 5 min at 72 °C [84]. The PCR products were assessed in 2% agarose gel electrophoresis (Akhtarian, Tehran, Iran) alongside with 50 bp DNA ladder (GenRuler, Thermo Fisher Scientific, USA, CA). To detect DNA fragments, DNA Green Viewer (Pars Tous, Iran, Mashhad) was applied. Then, visualization was done under UV light using Gel Documentation (ATP, Iran, Tehran).

**Table 3:** Characteristics of oligonucleotides used for *Echinococcus granulosus s.l.* complex multiplex PCR [84]

Primer name	End concentration (μM)	Sequence (5′–3′)	Product size (bp)
Echi Rpb2 F	1	TTGACCAAAGAAATCAGAC	1232
Echi Rpb2 R	1	CGCAAATACTCCATGG	
E.g complex F	0.15	TGGTCGTCTTAATCATTTG	110
E.g complex R	0.15	CCACAACAATAGGCATAA	
E.g ss cal F	2	CAATTTACGGTAAAGCAT	1001
E.g ss cal R	2	CCTCATCTCCACTCTCT	
E.g ss Ef1a F	1	TCCTAACATGCCTTGGTAT	706
E.g ss Ef1a R	1	GTTACAGCCTTGATCACG	
E.eq cal F	2	GCTTATTTAGGATCCCA	426
E.eq cal R	2	TCGTTTTTGCCAGTG	
E.eq coxI F	0.2	GTTGGGTTGGATGTT	124
E.eq coxI R	0.2	CAAAACAGGATCACTCTT	
E.ortp ATP6 F	0.05	GTGTCGTGTGTTTAGTGAG	1041
E.ortp ATP6 R	0.05	GCACTGATACAGGTGTTATT	
E.ortp CoxI F	0.2	GGTTTTATGGGTTGTTA	250
E.ortp CoxI R	0.2	ACACCACCAAACGTG	
E.cnd G6/G7 pold F	1	GGCCTTCATCTCCATAATA	617
E.cnd G6/G7 pold R	1	ATGAAGAGTTTGAAACTAAAG	
E.cnd G6/G7 NDI F	0.3	CTGCAGAGGTTTGCC	339
E.cnd G6/G7 NDI R	0.3	CACAACAGCATAAAGCG	
E.cnd G8/G10 Elp F	1.5	CCTAGTCTTCCCATGATA	283
E.cnd G8/G10 Elp R	1.5	ACAGAAGGCATATCCA	

### Sequencing

To verify the multiplex PCR resulting from genotyping, 59 samples were selected to sequence the gene target of *cox* 1 randomly from the cysts obtained from all various species of animals. The PCR reaction was conducted using a thermal cycler (ABI, USA) in a 20 μl final volume containing 50–100 ng gDNA, 0.2 mM dNTPs (Ampliqon, Denmark), 1.5 mM MgCl_2_ (Ampliqon, Denmark), 10 pmol of each primer (ordered from Pishgam Company), 1.5 U *Taq* DNA polymerase, and ddH_2_O up to the final volume. Amplification was done using the specific primer pair for *cox* 1 by JB3: 5′-TTTTTTGGGCATCCTGAGGTTTAT-3′ and JB4.5: 5′-TAAAGAAAGAACATAATGAAAATG-3′ [84] resulted in an amplicon fragment of 450 bp in length. The temperature conditions were an initial denaturation at 94 °C for 5 min; then 35 cycles of denaturation at 94 °C for 45 s, annealing at 57 °C for 45 s, and elongation at 72 °C for 45 s. The final extension was done at 72 °C for 5 min. PCR products were detected in 1% agarose gel electrophoresis (Akhtarian, Tehran, Iran). The PCR product was excised from agarose gel, and sent to the Company (Pishgam Company, Tehran, Iran) for purification and sequencing. The sequencing results were analyzed using BLAST.

### Phylogenetic analysis

Sequence data of *E. granulosus s.l.* isolates were complete alignment using the T-COFFEE software [85]. Also haplotypes defined by combination of single nucleotide polymorphisms were assigned using DnaSP V.6. Phylogenetic analysis were inferred from DNA sequences using Maximum Likelihood (ML) estimates with MEGAX based on Tamura-Nei model with 1000 bootstrap replicates. All positions containing gaps and missing data were eliminated.

### Statistical analysis

The obtained data were analyzed using SPSS version 16.0. In the first step, the data were entered into the software. Then, descriptive data analysis was performed, including frequency and prevalence calculation. The relationship between infection in animals with sex, age, cyst location, and fertility, and viability of infected animal species were analyzed using the chi-square test. With confidence level of 95%, *P-value* < 0.05 was considered as significant.

## Data Availability

The datasets used and analyzed during the current study are available from the corresponding author on reasonable request. All the sequences were deposited in the NCBI GenBank (https://www.ncbi.nlm.nih.gov).

## Abbreviations

CE: Cystic echinococcosis
PBS: Phosphate-buffered salin
gDNA: Genomic DNA
ML: Maximum likelihood
